# Combined effect of hyperhomocysteinemia and smoking on the severity of coronary artery disease in young adults ≤ 35 years of age: a hospital-based observational study

**DOI:** 10.1186/s12872-021-02302-0

**Published:** 2021-10-09

**Authors:** Jiayin Sun, Wei Han, Sijing Wu, Shuo Jia, Zhenxian Yan, Yonghe Guo, Yingxin Zhao, Yujie Zhou, Xiaoli Liu

**Affiliations:** Department of Cardiology, Beijing Anzhen Hospital, Capital Medical University, Beijing Institute of Heart Lung and Blood Vessel Disease, Beijing, 100029 China

**Keywords:** Coronary artery disease, Hyperhomocysteinemia, Smoking, Severity, Young

## Abstract

**Background:**

The prevalence of coronary artery disease (CAD) continues to increase among young Chinese adults. Current smoking has been recognized as a major risk factor for premature CAD, and hyperhomocysteinaemia (HHcy) has also been suggested to be associated with CAD progression. However, the combined effect of current smoking and HHcy on the severity of coronary artery stenosis in young adults is still uncertain.

**Methods:**

We consecutively collected young patients (18–35 years of age), diagnosed with CAD and underwent coronary angiography (CAG) at Anzhen Hospital between January 2013 and May 2020. HHcy was defined as serum homocysteine (Hcy) level > 15 µmol/L. The severity of coronary artery stenosis was evaluated by Gensini Score. The co-effect of current smoking and HHcy on CAD severity as well as the relationship between plasma Hcy, pack-years of smoking and CAD severity were assessed by multivariate linear regression analysis.

**Results:**

A total of 989 participants (mean age, 33 years; 96.2% male) fulfilling the criteria were enrolled in this study. Patients with both HHcy and current smoking accounted for 39.1% of all the subjects. Multivariate liner analysis indicated both serum Hcy levels (*β* 0.302; 95% CI 0.141–0.462; *P* < 0.001) and pack-years of smoking (*β* 0.523; 95% CI 0.265–0.781; *P* < 0.001) were independently associated with the severity of coronary artery stenosis after adjusting for other traditional confounders. In addition, serum Hcy levels were correlated with pack-years of smoking in young CAD patients (*r* = 0.116, *P* = 0.001). Moreover, combination of HHcy and current smoking was suggested to have higher risk for CAD severity (*β* 17.892; 95% CI 11.314–24.469; *P* < 0.001), compared with HHcy (*β* 7.471; 95% CI 0.009–14.934; *P* = 0.048) or current smoking (*β* 7.421; 95% CI 0.608–14.233; *P* = 0.033) alone.

**Conclusion:**

Combination of HHcy and smoking is independently associated with the severity of CAD in young patients ≤ 35 years of age.

## Introduction

Coronary artery disease (CAD), with a continuously rising prevalence and mortality, has become a significant public health problem in China [1]. Due to the changes in lifestyle, including the increased obesity rate and reduced physical exercise, the onset age for CAD has gradually gone down, while young CAD patients with onset age ≤ 45 years has gradually increased [2, 3]. Several traditional risk factors, such as age, current smoking status, diabetes mellitus (DM), and increased low-density lipoprotein cholesterol (LDL-C) level, have been identified to be correlated with the severity of CAD among young patients [4, 5]. Current smoking, considering as the most frequent and one of the most important risk factors for CAD in young adults, exerts a deleterious impact via impairing endothelial function. Moreover, some studies indicated hyperhomocysteinemia (HHcy), as a non-traditional risk factor, was positively associated with severe CAD by increasing lesion counts in patients with premature CAD, which was also due to the vascular endothelial damage and dysfunction [6]. In addition, previous studies have investigated the relationship between plasma Hcy and smoking. Cigarette smoking was showed to increase serum Hcy levels and decrease folate and vitamin B12 levels, which was strongly correlated with the duration of use and the number of cigarettes consumed [7]. Another study also indicated pack-years of smoking were significantly positively correlated with serum Hcy levels, which may due to the effect of oxidative damage [8].

Considering the adverse effects of HHcy and smoking exerting on endothelial function and atherosclerotic disease, along with the obvious correlation between the two factors, investigating the co-effect of HHcy and current smoking on the severity of premature CAD is demanded. Thus, the current study was conducted to determine the following: (1) the combined effect of HHcy and current smoking on the severity of coronary artery stenosis (assessed with a Ginsini scoring system) in young adults ≤ 35 years of age; (2) the association between CAD severity and both serum Hcy levels and pack-years of smoking in the study population.

## Methods

### Study population

In this observational study, we consecutively collected young patients (18–35 years of age), diagnosed with CAD and underwent coronary angiography (CAG) at Anzhen Hospital between January 2013 and May 2020. The exclusion criteria were as follows: repeated hospitalization, missing homocysteine data, and smoking cessation for more than1 year. Patients with severe renal insufficiency, hypothyroidism, psoriasis, multiple arteritis, Kawasaki disease, rheumatic heart disease, myocarditis, infective endocarditis, congenital heart disease, cardiomyopathy, valvular heart disease, or having vitamin or folate supplementation within three months were also excluded. This study was approved by the Institutional Ethics Committee of Beijing Anzhen Hospital. The demographic and clinical data we used were retrospectively obtained from electronic medical records.

### Definitions and grouping

Serum levels of Hcy > 15umol/L was defined as HHcy [9]. Occasional or regular smoking ≥ 1cig/day was defined as smoking status, which also included former smokers with cessation period ≤ 1 year [10]. Pack-years of smoking was defined as the average number of packs of cigarettes smoked per day multiplying the number of years of smoking. Hypertension was defined as a systolic blood pressure (SBP) ≥ 140 mmHg and/or diastolic blood pressure (DBP) ≥ 90 mmHg, or using antihypertensive medications currently [11]. Diabetic mellitus (DM) was defined as fasting blood glucose (FBG) ≥ 7.0 mmol/L and/or random glucose level ≥ 11.1 mmol/L, or previously diagnosed DM with the treatment of diet, oral agents, or insulin [12]. Hypertriglyceridemia was defined as triglycerides (TG) ≥ 1.7 mmol/L, hypercholesterolemia was defined as total cholesterol (TC) ≥ 5.2 mmol/L, a high low-density lipoprotein cholesterol (LDL-C) level was defined as LDL-C ≥ 3.4 mmol/L, and a low high-density lipoprotein cholesterol (HDL-C) level was defined as HDL-C < 1.0 mmol/L [13]. Serum uric acid (UA) level ≥ 420 mmol/L in males and ≥ 357 mmol/L in females was considered to be hyperuricemia [14]. Someone with average alcohol intake ≥ 50 g/day was considered to be drinker.

The study population was divided into four groups according to presence or absence of HHcy and smoking status, which were as follows: HHcy − Smoker − group, HHcy+ Smoker− group, HHcy−Smoker + group and HHcy+Smoker+ group.

All patients received coronary angiography using a standard technique. A luminal diameter stenosis ≥ 50% in any of the major coronary arteries, including the left main, left circumflex, left anterior descending, right coronary artery, and main branches with a diameter of more than 2.0 mm, was defined as CAD. In addition, patients who diagnosed as acute myocardial infraction were also considered to have CAD. Left main stenosis ≥ 50% was considered as a double-vessel disease. Multivessel disease was defined as ≥ 50% stenosis in more than one major coronary vessels. Gensini Score was used to quantify the severity of CAD [15]. Based on the results of CAG, the score of each lesion was calculated by severity score, which reflected the stenosis degree of luminal narrowing and modified by the collateral adjustment factor, multiplying region factor, which reflected the geographic importance of the lesion location in the coronary tree. The final Gensini Score was expressed as the sum of all the lesion scores.

### Data collection

Baseline venous blood samples were taken from all participants after an overnight fast within the first 24 h of hospitalization. The following biochemical parameters were analyzed: TG, TC, LDL-C, HDL-C, UA, blood urea nitrogen (BUN), creatinine (CR), glycated hemoglobin (HbA1c) and high-sensitivity C- reactive protein (hs-CRP). Hcy commercial kit (enzymatic cycling method) was used to test serum Hcy levels by a Beckman Coulter AU5400 automatic biochemical analyzer.

The clinical data of participants, including age, gender, body mass index (BMI), history of hypertension and DM, family history of CAD as well as smoking and drinking status, were retrospectively obtained from electronic medical records.

### Statistical analysis

All the analysis was conducted by the statistical software SPSS 22.0 (IBM-SPSS Inc., Chicago, USA). Kolmogorov–Smirnov test was used to evaluate the normality of continuous data. Accordingly, normally distributed continuous variables were presented as mean ± standard deviation, and non-normally distributed data as median [interquartile (25th–75th percentiles) range]. Where indicated, one-way analysis of variance and Kruskal–Wallis H test were applied to evaluate statistical differences among groups with different Hcy levels and smoking status. Then, pairwise comparison was performed among groups using S–N–K test or Mann–Whitney U test. Categorical variables were expressed as counts and percentages (%), and differences among these groups were examined by Chi-square test. The relationship between serum Hcy levels and pack-years of smoking was evaluated using Spearman analysis. Variables with a *P* value < 0.1 in the Univariate liner regression analysis as well as the traditional risk factors for CAD were selected and added into multivariate liner regression analysis to determine their effects on the severity of CAD, which was calculated by Beta with 95% confidence intervals (95% CI). Moreover, the relationship of Hcy, pack-years of smoking and the severity of CAD was also assessed by multivariate linear regression analysis. *P* values of < 0.05 in a two-sided test was considered statistically significant.

## Results

### Baseline clinical characteristics

**Fig. 1:**
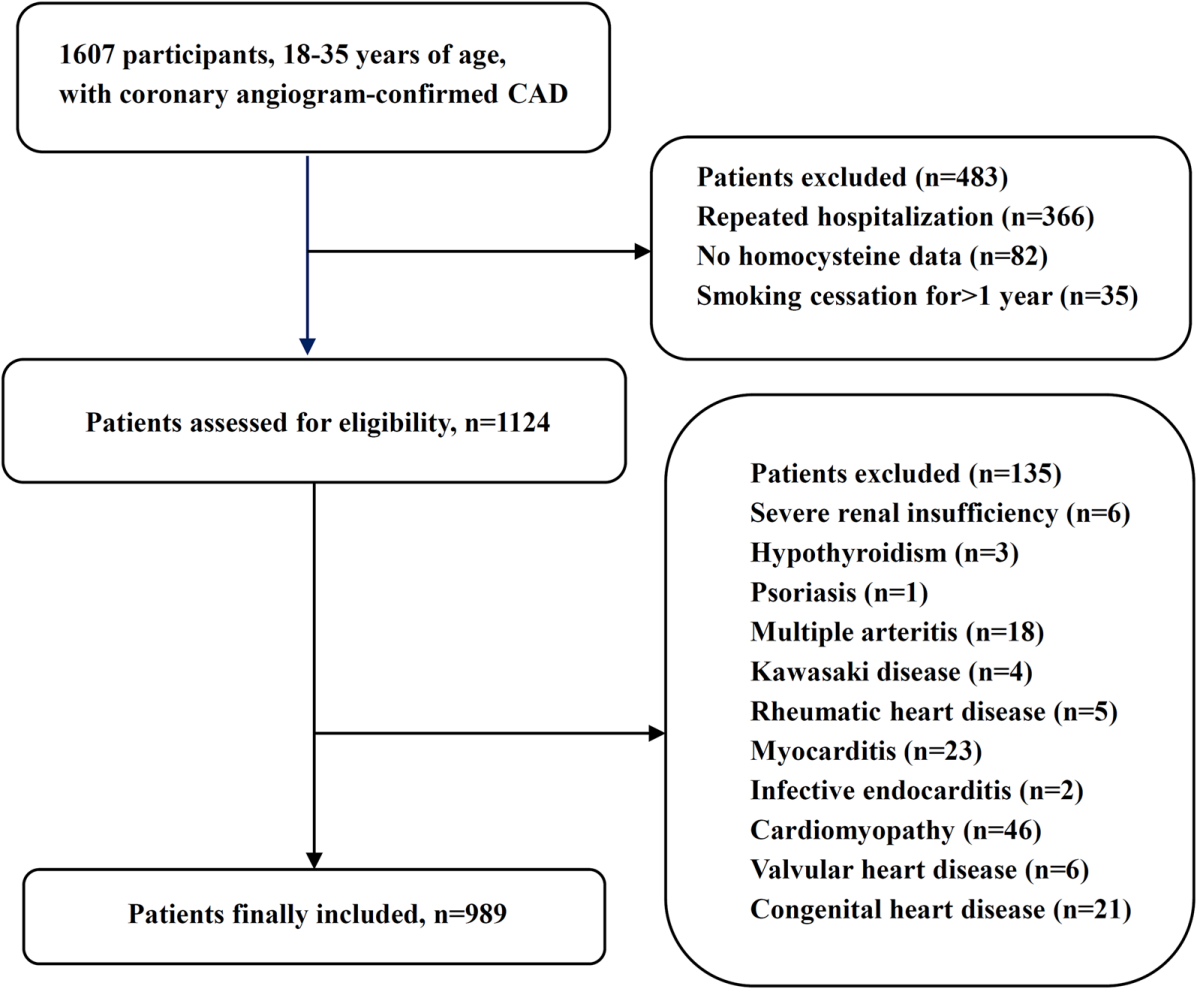
Flow chart illustrating the process of a participant enrolled in the study

As shown in Fig. 1, a total of 989 participants fulfilling the criteria were enrolled in this study (mean age, 33 years; 96.2% male). The baseline characteristics and biochemical findings of all the patients in four groups are listed in Table 1. Patients with HHcy accounted for 55.3% and patients with current smoking accounted for 68.4%, while patients with both HHcy and current smoking accounted for 39.1% of all the young participants. Smokers (both in HHcy−Smoker+ group and HHcy+Smoker+group) had a higher prevalence of male gender, drinker, hypertriglyceridemia and family history of CAD, as well as increased levels of TG and hs-CRP compared with non-smokers (both in HHcy−Smoker− group and HHcy+Smoker− group). Meanwhile, the HHcy+Smoker− group also had higher male prevalence and elevated hs-CRP levels compared with the HHcy−Smoker− group, but not as significant as smokers. Moreover, patients with HHcy (both in HHcy+Smoker− group and HHcy+Smoker+group) had increased levels of CR and UA, but a lower prevalence of DM compared with patients without HHcy (both in HHcy−Smoker− group and HHcy−Smoker+group).

**Table 1:** Baseline clinical characteristics of study population

Characteristics	Total (n = 989)	HHcy−Smoker− (n = 153)	HHcy+Smoker− (n = 160)	HHcy−Smoker+(n = 289)	HHcy+Smoker+(n = 387)
Age (years)	33 (30–34)	33 (30–34)	32 (29–34)	33 (31–34)	32 (30–34)
Male, n (%)	951 (96.16)	128 (83.66)	151 (94.38)^a^	288 (99.65)^ab^	384 (99.22)^ab^
SBP (mmHg)	126.44 ± 15.61	126.87 ± 14.97	127.44 ± 14.94	127.36 ± 16.71	125.18 ± 15.25
DBP (mmHg)	78.01 ± 13.19	78.21 ± 14.18	78.43 ± 14.13	78.25 ± 13.71	77.57 ± 11.97
Heart rate (bpm)	75.94 ± 11.71	75.34 ± 10.17	77.25 ± 11.36	76.21 ± 12.14	75.43 ± 12.07
Drinker, n (%)	182 (18.40)	14 (9.15)	9 (5.63)	80 (27.68)^ab^	79 (20.41)^ab^
*Medical history and coronary risk factors*					
BMI (kg/m^2^)	28.34 ± 5.01	27.68 ± 6.15	28.09 ± 4.57	28.72 ± 5.08	28.44 ± 4.57
Hypertension, n (%)	485 (49.04)	74 (48.37)	77 (48.13)	149 (51.56)	185 (47.80)
Diabetes mellitus, n (%)	191 (19.31)	36 (23.53)	25 (15.63)	82 (28.37)^b^	48 (12.40)^ac^
Hypertriglyceridemia, n (%)	559 (56.52)	65 (42.48)	82 (51.25)	178 (61.59)^a^	234 (60.47)^a^
Hypercholesterolemia, n (%)	336 (33.97)	51 (33.33)	45 (28.13)	114 (39.45)	126 (32.56)
High LDL-C, n (%)	307 (31.04)	37 (24.18)	42 (26.25)	104 (35.99)	124 (32.04)
Low HDL-C, n (%)	677 (68.45)	96 (62.75)	106 (66.25)	200 (69.20)	275 (71.06)
Family history of CAD, n (%)	127 (12.84)	13 (8.50)	8 (5.00)	42 (14.53)^b^	64 (16.54)^b^
Hyperuricemia, n (%)	437 (44.19)	61 (39.87)	71 (44.38)	119 (41.18)	186 (48.06)
Prior stroke, n (%)	5 (0.51)	0 (0.00)	0 (0.00)	3 (1.00)	2 (0.50)
*Laboratory results*					
BUN (mmol/L)	4.73 ± 1.31	4.67 ± 1.46	4.77 ± 1.31	4.66 ± 1.17	4.79 ± 1.31
CR (µmol/L)	74.78 ± 15.12	72.21 ± 14.81	77.64 ± 19.96^a^	72.58 ± 12.39^b^	76.24 ± 14.39^ac^
HbA1c (%)	5.6 (5.3–6.2)	5.6 (5.2–6.7)	5.5 (5.2–6.1)	5.7 (5.4–6.8)^b^	5.5 (5.2–5.9)^c^
TG (mmol/L)	1.88 (1.30–2.85)	1.47 (1.01–2.25)	1.75 (1.15–2.32)	2.05 (1.39–3.36)^ab^	2.02 (1.43–2.93)^ab^
TC (mmol/L)	4.74 ± 1.73	4.64 ± 2.10	4.66 ± 2.01	4.92 ± 1.67	4.69 ± 1.46
HDL-C (mmol/L)	0.92 ± 0.25	0.95 ± 0.23	0.93 ± 0.21	0.92 ± 0.35	0.90 ± 0.19
LDL-C (mmol/L)	3.03 ± 1.56	2.95 ± 1.97	3.01 ± 1.91	3.13 ± 1.49	2.99 ± 1.23
UA (µmol/L)	411.23 ± 97.88	390.73 ± 101.55	417.69 ± 107.07^a^	405.44 ± 83.03	420.99 ± 101.54^a^
Hcy (µmol/L)	15.60 (11.30–26.60)	10.30 (8.70–12.20)	22.95 (16.53–33.38)^a^	11.00 (9.00–13.10)^ab^	25.80 (17.80–39.40)^abc^
hs-CRP (mg/L)	2.70 (0.97–8.93)	1.37 (0.54–4.85)	2.55 (0.97–6.10)^a^	3.11 (1.27–11.29)^ab^	3.12 (0.95–12.91)^ab^

Data are expressed as mean ± standard deviation, medians with interquartile range or number (%)

*CAD* coronary artery disease, *HHcy* hyperhomocysteinemia, *SBP* systolic blood pressure, *DBP* diastolic blood pressure, *BMI* body mass index, *BUN* blood urea nitrogen, *CR* creatinine, *HbA1c* glycated hemoglobin, *TG* triglyceride, *TC* total cholesterol, *HDL-C* high-density lipoprotein cholesterol, *LDL-C* low-density lipoprotein cholesterol, *UA* uric acid, *hs-CRP* high-sensitivity C-reactive protein

^a^*p* < 0.05 versus HHcy−Smoker−

^b^*p* < 0.05 versus HHcy+Smoker−

^c^*p* < 0.05 versus HHcy−Smoker+

In addition, stable coronary artery disease (SCAD) diagnosis and drug treatment were more prevalent in patients of HHcy−Smoker− group, while AMI diagnosis and coronary intervention were more prevalent in the counterparts of the other three groups. Analysis of the CAG findings demonstrated that there was no difference on the numbers of lesion vessels among four groups, except for having a smaller proportion of patients with no lesion vessels in the HHcy+Smoker+ group. Besides, the highest value of Gensini Score was observed in the HHcy+Smoker+group, while the lowest was found in the HHcy−Smoker− group [44 (24–78) in HHcy+Smoker+group vs. 32 (18.5–50) in HHcy−Smoker+group or 32 (13–57.5) in HHcy+Smoker− group vs. 24 (11–43) in HHcy−Smoker− group, *P* < 0.05 respectively]. Patients in the HHcy+Smoker+group also had a relatively lower LVEF level (Table 2).

**Table 2:** Clinical diagnosis, angiographic findings, medical treatment and cardiac function of study population

Characteristics	Total (n = 989)	HHcy−Smoker− (n = 153)	HHcy+Smoker− (n = 160)	HHcy−Smoker+ (n = 289)	HHcy+Smoker+ (n = 387)
*Diagnosis*					
SCAD	102 (10.31)	32 (20.92)	13 (8.13)^a^	33 (11.42)^a^	24 (6.20)^a^
UAP	379 (38.32)	74 (48.37)	67 (41.88)	97 (33.56)^a^	141 (36.43)
AMI	508 (51.37)	47 (30.72)	80 (50.00)^a^	159 (55.02)^a^	222 (57.36)^a^
*Angiographic findings of vessel involvement*					
None	48 (4.85)	17 (11.11)	11 (6.88)	16 (5.54)	4 (1.03)^abc^
Single vessel	447 (45.20)	66 (43.14)	70 (43.75)	134 (46.37)	177 (45.74)
Double vessel	236 (23.86)	40 (26.14)	37 (23.13)	60 (20.76)	99 (25.58)
Triple vessel	258 (26.09)	30 (19.61)	42 (26.25)	79 (27.33)	107 (27.65)
Multi-vessel	494 (49.95)	70 (45.75)	79 (49.38)	139 (48.1)	206 (53.23)
*Treatment*					
Drug	211 (21.33)	51 (33.33)	33 (20.63)^a^	54 (18.69)^a^	73 (18.86)^a^
Intervention	754 (76.24)	100 (65.36)	124 (77.50)^a^	228 (78.89)^a^	302 (78.04)^a^
Coronary artery bypass grafting	24 (2.43)	2 (1.31)	3 (1.87)	7 (2.42)	12 (3.10)
*Cardiac function*					
LVEF	60 (55–66)	62 (58–67)	61 (53–66)	61 (55–66)	60 (53–65)^ac^
Gensini Score	34 (20–62)	24 (11–43)	32 (13–57.5)^a^	32 (18.5–50)^a^	44 (24–78)^abc^

Data are expressed as the number (%)

*SCAD* stable coronary artery disease, *UAP* unstable angina pectoris, *AMI* acute myocardial infraction, *HHcy* hyperhomocysteinemia, *LVEF* left ventricular ejection fraction

^a^*p* < 0.05 versus HHcy−Smoker−

^b^*p* < 0.05 versus HHcy+Smoker−

^c^*p* < 0.05 versus HHcy−Smoker +

### Univariate analysis of different CAD risk factors

As shown in Table 3, traditional CAD risk factors such as current smoker status, hypertriglyceridemia, High LDL-C and low HDL-C were apparently associated with the severity of CAD (*P* < 0.05), while drinker showed a negative correlation. On the contrary, univariate analysis also showed that age, male gender, BMI, hypertension, DM, hypercholesterolemia and family history of CAD had no significant association with the severity of CAD. Moreover, non-traditional risk factors, such as UA, especially HHcy, were also obviously related to the severity of CAD (for HHcy, *β* 11.463; 95% CI 7.496–15.429; *P* < 0.001).

**Table 3:** Univariate liner regression analysis of the severity of CAD with variables

Variables	*β*	95% *Cl*	*P*
Age	− 0.406	− 1.097, 0.284	0.249
Male	6.102	− 4.304 to 16.509	0.250
BMI	0.002	− 0.436 to 0.440	0.993
Drinker	− 6.805	− 11.952 to − 1.657	**0.01**
Smoker	9.575	5.321–13.829	** < 0.001**
Hypertension	0.878	− 3.126 to 4.881	0.667
Diabetes mellitus	3.696	− 1.369 to 8.762	0.152
Hypertriglyceridemia	4.686	0.659–8.713	**0.023**
Hypercholesterolemia	4.127	− 0.091 to 8.346	0.055
High LDL-C	8.185	3.890–12.481	** < 0.001**
Low HDL-C	4.304	0.005–8.603	**0.049**
Family history of CAD	0.294	− 5.698 to 1.973	0.206
Hyperhomocysteinemia	11.463	7.496–15.429	** < 0.001**
Uric acid	0.026	0.006–0.047	**0.013**
BUN	0.025	− 0.056 to 0.107	0.543
CR	0.057	− 0.012 to 0.125	0.105

Bold values indicate statistical significance

*CAD* coronary artery disease, *BMI* body mass index, *HDL-C* high-density lipoprotein cholesterol, *LDL-C* low-density lipoprotein cholesterol, *BUN* blood urea nitrogen, *CR* creatinine, CI confidence interval

### Multivariate liner regression analysis of different CAD risk factors

**Fig. 2:**
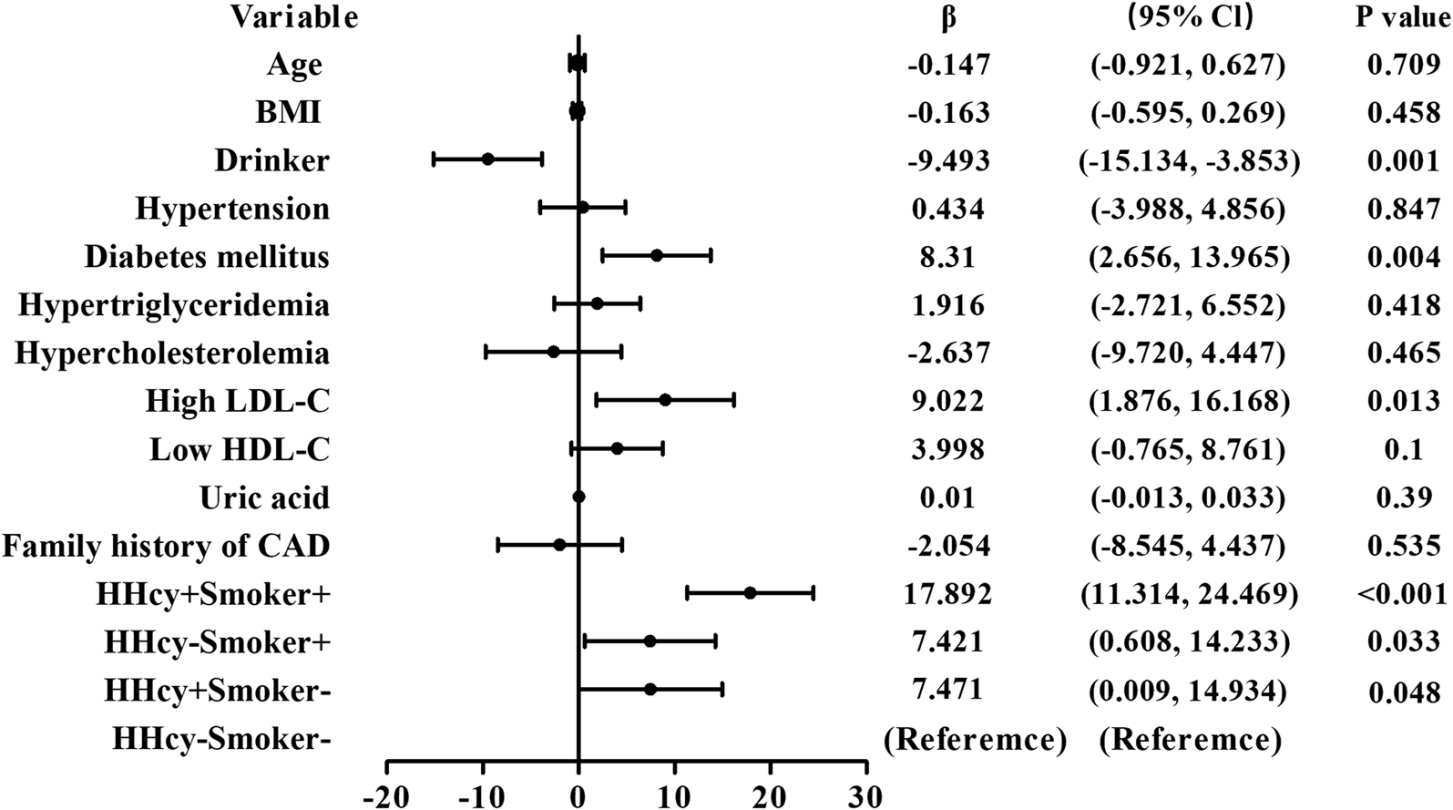
Forest plot of multivariate linear regression analysis of the severity of CAD with different CAD risk factors. *CAD* coronary artery disease, *BMI* body mass index, *HDL-C* high-density lipoprotein cholesterol, *LDL-C* low-density lipoprotein cholesterol, *HHcy* hyperhomocysteine, CI confidence interval

Except for the variables with *P* < 0.1in the univariate analysis, traditional CAD risk factors with *P* > 0.1, including age, BMI, hypertension, DM and family history of CAD were also added in the Multivariate liner regression analysis. The results indicated that DM (*β* 8.310; 95% CI 2.656–13.965; *P* = 0.004) and high LDL-C (*β* 9.022; 95% CI 1.876–16.168; *P* = 0.013) were the significant risk factors, while drinker was a protective factor for the severity of CAD among young adults, after adjusting for confounding factors. Moreover, patients with both HHcy and smoking had 17.892-fold risk for CAD severity (95% CI 11.314–24.469) compared with those without HHcy and smoking, and had more than two times risk for the severity of CAD, as compared to patients with either HHcy or smoking (for HHcy−Smoker+, *β* 7.421; 95% CI 0.608–14.233; for HHcy+ Smoker−, *β* 7.471; 95% CI 0.009–14.934) (Fig. 2).

### Correlation of serum Hcy levels with Pack-years of smoking in young CAD patients

**Fig. 3:**
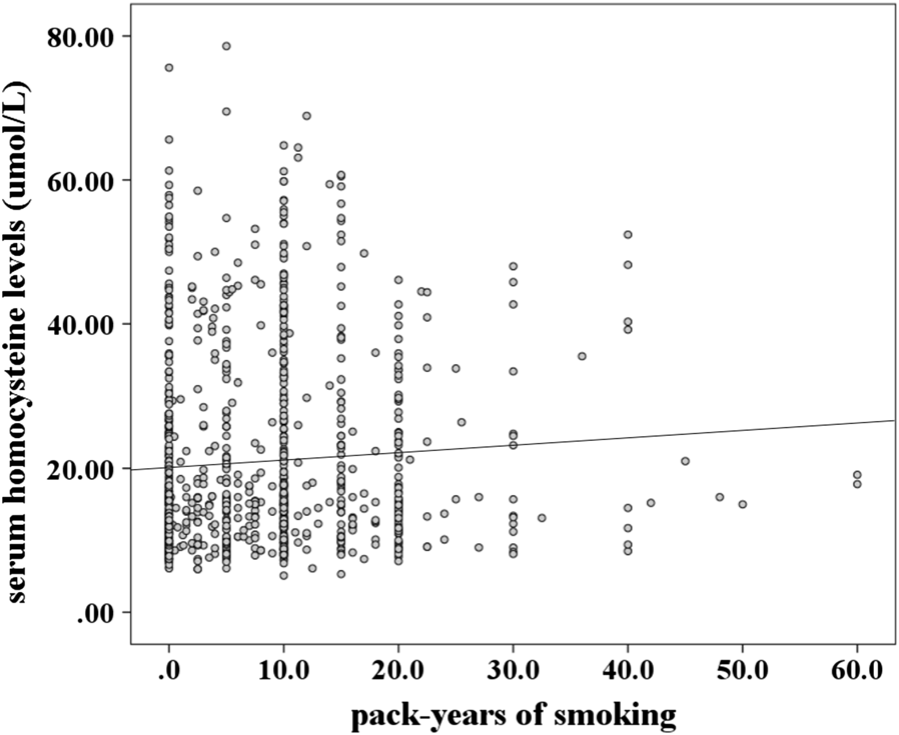
Correlation of serum homocysteine levels with pack-years of smoking (*r* = 0.116, *P* = 0.001)

Figure 3 showed that serum Hcy levels were apparently correlated with pack-years of smoking in young CAD patients (*r* = 0.116, *P* = 0.001).

### Multivariate analysis of CAD severity with serum Hcy levels and pack-years of smoking

Multivariate liner regression analysis was performed to determine the correlation of CAD severity and serum Hcy levels as well as pack-years of smoking. As shown in Table 4, after adjusting for BMI, drinker, hypertension, DM, triglyceride, HDL-C and LDL-C, both serum Hcy levels (*β* 0.302; 95% CI 0.141–0.462; *P* < 0.001) and pack-years of smoking (*β* 0.523; 95% CI 0.265–0.781; *P* < 0.001) were significantly associated with the severity of CAD in young adults.

**Table 4:** Multivariate analysis of the severity of CAD with homocysteine and pack-years of smoking

	Model 1^a^			Model 1^b^		
Variables	*β*	95% *Cl*	*P*	*β*	95% *Cl*	*P*
Pack-years of smoking	0.398	0.167, 0.428	**0.001**	0.523	0.265, 0.781	**< 0.001**
Homocysteine	0.316	0.170, 0.462	**< 0.001**	0.302	0.141, 0.462	**< 0.001**

Bold values indicate statistical significance

^a^No other risk factors were adjusted

^b^Adjusted for body mass index (BMI), drinker, Hypertension, Diabetes mellitus, triglyceride, high-density lipoprotein cholesterol (HDL-C), and low-density lipoprotein cholesterol (LDL-C)

## Discussion

To our knowledge, this observational study firstly investigated the co-effect of HHcy and current smoking on the CAD severity in patients less than 35 years of age. From this large-scale study, we found that the combination of HHcy and smoking, acting as the most important risk factor, was independently associated with the severity of coronary artery stenosis among young subjects.

Young patients with CAD represent a minority proportion, about 6–10% adults below 40 years of age are diagnosed with CAD, but the incidence has been obviously increased [16]. Among the variety of conventional risk factors involved in the progression of premature CAD, current smoking was considered to be the most frequent one, which exerted significant deleterious effect [17]. The amount of cigarette smoking, especially the pack-years of smoking, have been shown to be associated with vascular disease. A study involving 416 middle-aged and elderly patients showed Pack-years might be the most important factor that was correlated with the severity of coronary stenosis in terms of Gensini Score (*r* = 0.193, *P* = 0.013) [18]. As for younger subjects, Cheezum et al. [19] enrolled a population with a median age 50 years and suggested a dose-dependent (Pack-years ≥ 12) association between smoking and extent of plaque. In the current study, we firstly indicated a positive correlation between pack-years of smoking and severity of coronary atherosclerosis in young patients ≤ 35 years of age, which was independent of the other conventional risk factors (*β* 0.523; 95% *CI* 0.265–0.781; *P* < 0.001). Furthermore, the risk due to smoking was shown to last up to 20 years for CAD after smoking cessation, but since 1 year cessation was significantly associated with 2% lower risk for CAD on average [20], young patients with smoking cessation for more than1 year were excluded in the study.

Hcy level is a well-known biomarker of CAD and elevated concentrations of Hcy have been identified to be a crucial promoter for atherosclerotic vascular disease. However, some controversy exists regarding the causal role of Hcy in CAD pathogenesis, since lowering Hcy levels in patients with CAD has not shown any benefit and common genetic variants that influence Hcy levels are not associated with risk of CAD [21, 22]. Nevertheless, several observational studies found HHcy, as an important marker, was significantly associated with severe CAD in younger patients [6, 23]. In this study, the relationship between concentrations of Hcy and the severity of coronary artery stenosis was also investigated, which showed that serum Hcy levels were positively associated with angiographic severity expressed by Gensini Score (*β* 0.302; 95% CI 0.141–0.462; *P* < 0.001). Our data were consistent with the results of previous study conducted by Karadeniz and colleagues [24], which enrolled 503 elderly patients with acute coronary syndrome, and suggested Hcy levels were independently related to the severity of CAD (*β* 0.144; *P* = 0.017). However, the Karadeniz et al. [24] study showed no significant effect of smoking on atherosclerotic plaque progression, which was not the same as ours, considering the differences in age and health status of the study participants.

In our study, after eliminating the impact of other CAD risk factors, both serum Hcy levels as well as pack-years of smoking were independently associated with Gensini Score, which indicated combined consideration of Hcy levels and amount of smoking could better reflect the development of CAD. In addition, the results also suggested patients with both HHcy and current smoking had higher risk for CAD severity, compared with those without or with just one factor (HHcy or current smoking). Previous study analyzed the influences of smoking and Hcy levels on the degree of arteriolar retinopathy, which was associated with greater cardiovascular risk, and the results showed in subjects with grade II retinopathy, smoking was related to increased plasma Hcy levels and could independently contribute to the progression of arteriolar retinopathy [25], reflecting the positive effect of smoking and Hcy levels on microvascular disease. Moreover, we found young CAD patients with both HHcy and smoking had decreased value of LVEF, which might be due to the relatively increased Gensini Score and more myocardial ischemia.

Several possible mechanisms might explain the combined effect of HHcy and current smoking on the progression of CAD, since both of these risk factors could exert similar effects. (1) Nicotine and carbon monoxide in tobacco can produce direct vascular endothelial damage by reducing the production of nitric oxide (NO) and prostacyclin, and aggravating the inflammatory reaction [26]. Hcy also have a variety of toxic effects on the vasculature, including impairing endothelial function by decreasing the concentration of NO, inducing vascular remodeling by increasing the smooth muscle cells synthesis, and elevating adventitial inflammation [27]. (2) Both smoking and Hcy may exert a prothrombotic effect via increasing platelet aggregation and destroying the balance of coagulation and fibrinolysis system [26, 28]. (3) Smoking may promote dyslipidemia, while HHcy may cause Hcy-related LDL atherogenesis, such as small LDL particle size and its oxidative modification [26, 29]. Those all suggest a strong potential for interaction between two factors in the development of atherosclerosis. In addition, except for damaging the vascular tree independently, smoking and Hcy are also related. Studies have showed that smoking could exert a deleterious impact on plasma Hcy. The pack-years of smoking directly increased the serum Hcy levels through regulating Hcy metabolism by reducing serum folate and vitamin B12 concentrations, as well as activating the systemic oxidation reaction system and damaging the anti-oxidation defense system [7, 8], which indicated the potential effect of smoking on the progression of CAD among young subjects.

The clinical importance of this study is emphasizing the co-effect of current smoking and HHcy on the development and extent of premature CAD, which shows that the combination of these two risk factors has more effect on the severity of CAD among patients ≤ 35 years of age. From the perspective of CAD prevention, except for stressing the importance of smoking cessation, a young population should pay more attention to the adverse effect of HHcy, and have a healthy lifestyle, such as keeping weight, sufficient vitamin B and folic acid intake, to maintain Hcy levels within the normal range. In addition, serum Hcy levels are associated with smoking in a dose-dependent way, which suggests the potential benefits of smoking cessation in the prevention of CAD among young adults.

## Limitations

Our study has a few limitations. First, although plasma Hcy is mainly determined by vitamin B and folic acid intake, which was also affected by smoking, the levels of vitamin B and folic acid were not measured in this retrospective study. Second, since the majority of young CAD patients were male, the results had limited value for the young female population. Third, Gensini Score, rather than the Syntax Score, was used to evaluate the severity of CAD in the study, since the severity was assessed by CAG reports due to the long interval for participants enrollment. However, Gensini Score was identified to be none inferior to Syntax Score considering the relevance and equivalence [30].

## Conclusion

Combination of HHcy and smoking is independently associated with the severity of CAD in young patients ≤ 35 years of age.

## Data Availability

The datasets used and/or analyzed during the current study are available from the corresponding author on reasonable request.

## Abbreviations

CAD: Coronary artery disease
HHcy: Hyperhomocysteinemia
CAG: Coronary angiography
Hcy: Homocysteine
DM: Diabetes mellitus
LDL-C: Low-density lipoprotein cholesterol
SBP: Systolic blood pressure
DBP: Diastolic blood pressure
FBG: Fasting blood glucose
TG: Triglycerides
TC: Total cholesterol
HDL-C: High-density lipoprotein cholesterol
UA: Uric acid
BUN: Blood urea nitrogen
CR: Creatinine
HbA1c: Glycated hemoglobin
hs-CRP: High-sensitivity C-reactive protein
BMI: Body mass index
AMI: Acute myocardial infraction
UAP: Unstable angina pectoris
SCAD: Stable coronary artery disease
LVEF: Left ventricular ejection fraction
CI: Confidence intervals
